# Modified fibrin hydrogel for sustained delivery of RNAi lipopolyplexes in skeletal muscle

**DOI:** 10.1093/rb/rbac101

**Published:** 2022-12-13

**Authors:** Ellen Ngarande, Emma Doubell, Ousman Tamgue, Manuel Mano, Paul Human, Mauro Giacca, Neil Hamer Davies

**Affiliations:** Cardiovascular Research Unit, Department of Surgery, Faculty of Health Sciences, University of Cape Town, Observatory 7925, South Africa; Cardiovascular Research Unit, Department of Surgery, Faculty of Health Sciences, University of Cape Town, Observatory 7925, South Africa; International Centre for Genetic Engineering and Biotechnology (ICGEB), Cape Town Component, Observatory 7925, South Africa; Institute of Infectious Diseases and Molecular Medicine (IDM), Department of Pathology, Division of Immunology and South African Medical Research Council (SAMRC) Immunology of Infectious Diseases, Faculty of Health Sciences, University of Cape Town, Observatory 7925, South Africa; King’s College London, British Heart Foundation Centre of Research Excellence, School of Cardiovascular Medicine & Sciences, WC2R 2LS, London, UK; Cardiovascular Research Unit, Department of Surgery, Faculty of Health Sciences, University of Cape Town, Observatory 7925, South Africa; King’s College London, British Heart Foundation Centre of Research Excellence, School of Cardiovascular Medicine & Sciences, WC2R 2LS, London, UK; Cardiovascular Research Unit, Department of Surgery, Faculty of Health Sciences, University of Cape Town, Observatory 7925, South Africa

**Keywords:** RNA interference, controlled release, fibrin, lipopolyplex

## Abstract

RNA interference is a promising therapeutical approach presently hindered by delivery concerns such as rapid RNA degradation and targeting of individual tissues. Injectable hydrogels are one potentially simple and direct route towards overcoming these barriers. Here we report on the utility of a combination of a mildly modified form of the clinically utilised fibrin hydrogel with Invivofectamine^®^ 3.0, a lipid nonviral transfection vector, for local and sustained release. PEGylation of fibrin allowed for controlled release of small interfering RNA (siRNA)-lipopolyplexes for at least 10 days and greatly increased the stability of fibrin *in vitro* and *in vivo*. A 3D cell culture model and a release study showed transfection efficacy of siRNA-lipopolyplexes was retained for a minimum of 7 days. Injection in conjunction with PEGylated-fibrinogen significantly increased retention of siRNA-lipopolyplexes in mouse skeletal muscle and enhanced knockdown of myostatin mRNA that correlated with muscle growth. Thus, the increased efficacy observed here for the combination of a lipid nanoparticle, the only type of nonviral vector approved for the clinic, with fibrin, might allow for more rapid translation of injectable hydrogel-based RNA interference.

## Graphical abstract

**Figure:**
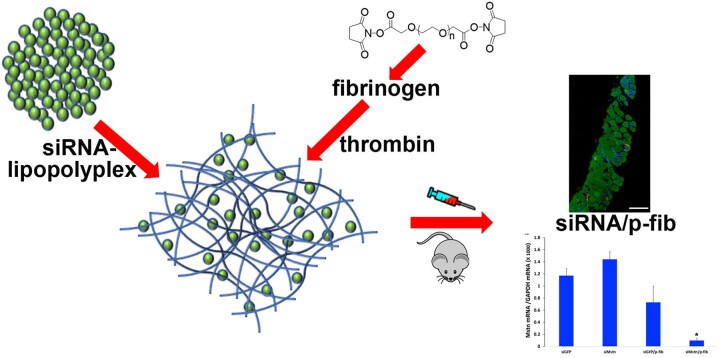

## Introduction

Small interfering RNA (siRNA) based therapeutics with their ability to specifically target the expression of any gene of interest have the potential to transform medicine. However, due to a number of impediments to their delivery *in vivo*, such as degradation by nucleases and a strong tendency to accumulate in organs such as the liver and lungs [1], their translation to the clinic is presently limited to two drugs, the first of which was patisiran, a lipid nanoparticle formulation containing siRNA that targets the expression of transthyretin by the liver [2]. A potential strategy to target other organs more effectively is the use of hydrogels as localized controlled release depots of siRNA [3, 4]. It is perhaps most desirable that the hydrogel scaffolds are injectable allowing for a minimally invasive approach and the siRNA either chemically modified or encapsulated within a nonviral nanoparticle delivery vehicle such that degradation is decreased, and cellular transfection augmented. A wide range of hydrogels and siRNA delivery vehicle combinations have been assessed for their ability to optimize delivery to targeted organs. Though, both synthetic and natural based hydrogels (e.g. polyethylene diacrylate hydrogels for delivery of si-Noggin to bone [5] and collagen for subcutaneous delivery of si-Interleukin-6 [6]) have been utilized, the natural hydrogel fibrin, with good potential for translation due to its FDA approval, has received very limited attention in the context of siRNA delivery.

Fibrin has been extensively investigated with respect to the delivery of growth factors [7–9], adenoviruses [10] and cell delivery [11] *in vivo*, but there is only one *in vitro* study to our knowledge that has examined siRNA delivery in the context of a fibrin hydrogel [12]. Knockdown of Noggin expression was observed when cationic lipofectamine 2000 complexed with Noggin siRNA was adsorbed onto preformed fibrin hydrogels. In a form of reverse transfection, cells were seeded onto the hydrogel surface decorated with lipoplexes thus ensuring direct contact. The study provided initial proof that fibrin could allow for RNAi agent delivery, though it was not designed to examine controlled release and only assessed the potential of fibrin as a nucleic acid delivery vehicle *in vitro*. One disadvantage of naturally derived polymers is that they lack adequate mechanical properties thereby compromising their application *in vivo* [13–15]. This aspect is of particular concern for the use of fibrin as it limits duration after implantation *in vivo* due to proteolytic degradation [16]. One relatively gentle modification that has been shown to limit proteolytic degradation *in vitro* is the crosslinking of fibrin with bivalent synthetic polyethylene glycol (PEG) molecules [17]. As PEG molecules are also FDA approved [18], it has been suggested that fibrin modified in this manner has greater potential for translation [19]. The value of PEGylating fibrin with respect to its potential as a controlled release scaffold has been demonstrated through the improved delivery of stem cells and growth factors *in vivo* [20, 21].

As indicated above, liposomal nanoparticle encapsulation of siRNA has shown the most progression and success in clinical application. Thus, here we have utilized the commercially available Invivofectamine^®^ 3.0, a cationic lipid transfection reagent developed to optimize siRNA delivery and minimize toxicity in preclinical *in vivo models* [22], to evaluate the potential of PEGylated fibrin (p-fibrin) as a controlled release scaffold for localized delivery of siRNA-lipopolyplexes in skeletal muscle. We have focussed on this approach for extending delivery as both the hydrogel and type of RNAi delivery vehicle used have high translation potential.

## Materials

Fibrinogen (human serum) and thrombin (bovine plasma) were obtained from Sigma Aldrich, MO, USA. PEG (Succinimidyl Carboxymethyl Ester)_2_ MW3500 (PEG-(SMC)_2_) was obtained from JenKem Technology, TX, USA. Alexa Fluor™ 647 conjugated fibrinogen (human plasma); Invivofectamine^®^ 3.0; RNAlater^®^ Solution; Alexa Fluor^®^ 660 C2-maleimide and ethidium homodimer-1 (EthD-1) stain from Live/Dead™ cell viability assay kit were obtained from Thermo Fisher Scientific, MA, USA. siRNA against green fluorescent protein (GFP) GCAUCAAGGUGAACUUCAAdTdT (sense) (siGFP) and scrambled negative control siRNA (siNegative) were obtained from Bioneer, Korea. Cy3-labelled siGFP (siGFP-Cy3), Cy3-labelled siRNA against myostatin (siMstn-Cy3): AAGAUGACGAUUAUCACGCUA-dTdT-Cy3 (sense) were obtained from GE Healthcare Dharmacon, CO, USA. AllStars Hs Cell Death Control siRNA 1027299 (Death siRNA); scrambled negative control siRNA AllStars negative siRNA (Neg siRNA control); PCR primers (see Supplementary Table S1) were obtained from Qiagen, Netherlands. Rabbit anti-GFP–ChIP Grade (AB290); rabbit anti-β-tubulin (AB6046) primary antibodies were obtained from Abcam, UK. Roche Transcriptor first strand cDNA synthesis kit, LightCycler^®^ 480 SYBR Green I master mix were obtained from Roche Diagnostics, Germany.

## Methods

### Fibrinogen PEGylation, Cy5 labelling and hydrogel formulation

PEG-SMC_2_ was incubated with fibrinogen (40 mg/ml Hepes buffered saline (HBS) pH 7.8) at a 5:1 molar ratio for 1 h at 37°C. For fibrin and PEGylated gels, final concentrations were: 10 mg/ml fibrinogen; 0.07 units/ml thrombin; 4.5 mM Ca^2+^ in HBS pH 7.4. siRNA-Invivofectamine^®^ lipopolyplexes were added to the pre-gel solution when required.

### Rheology

Small-strain oscillatory shear rheometry was performed on each gel type (*n* = 4) using a Kinexus Pro rheometer (Malvern Instruments, UK), with a 20 mm diameter upper flat plate geometry. Prior to polymerization, hydrogels were pipetted onto the preheated stage (37°C). The upper geometry was immediately lowered to a gap of 0.5 mm and enclosed in a 100% humidity environment. After 30 min gelling time, a frequency sweep from 0.1 to 10 Hz at 1% strain was conducted to measure the storage (*G*′) moduli. All hydrogels were compared at 1 Hz.

### Fibrin hydrogel stability in aqueous solution

Unmodified and PEGylated fibrin gels (50 µl) were polymerized and incubated in HBS pH 7.4 (150 µl) at 37°C with gentle agitation and the supernatant collected at 30 min, 1, 3, 5, 7, 10 days with buffer replacement after each time interval collection until the gels completely degraded as determined visually. Fibrin degradation products were quantified using Bradford reagent and a fibrinogen standard curve.

### siRNA release from unmodified and PEGylated fibrin gels

Unmodified and PEGylated fibrin gels containing 1 µg siRNA, (siNegative: see Supplementary Table S2 for all siRNA sequences) complexed in Invivofectamine^®^ 3.0 were prepared and incubated in HBS pH 7.4 at 37°C. Samples were collected as described above until fibrin gels had completely degraded. The siRNA released at each time point was quantified densitometrically after separation on a 2% (w/v) agarose gel and ethidium bromide staining. A fresh 5-point siRNA standard curve was included per gel run to allow for quantification. SDS (0.5% w/v final concentration) was added to an aliquot of the released samples to dissociate siRNA from the lipopolyplexes. siRNA bands were imaged and quantified using an InGENIUS3 documentation system (Syngene, India).

### Bioactivity of eluted lipopolyplexes

PEGylated fibrin gels (50 µl) containing 1 µg siNegative or siGFP-Cy3 complexed with Invivofectamine^®^ 3.0 were prepared under sterile conditions. Gels were polymerized and washed in 10% FBS in MCDB media for 30 min and then incubated at 37°C under sterile conditions in 10% FBS in MCDB media (200 µl) over a period of 7 days. Eluents were collected at days 2, 4 and 7. The entire eluent was immediately used to transfect HT1080 cells stably transfected with GFP (HT1080-GFP). Post transfection, cells were analysed for GFP expression using flow cytometry. HT1080-GFP cells were washed with warm PBS (pH 7.4) to remove media and collected after trypsinization. GFP fluorescence intensity was measured on a FACSCalibur flow cytometer (BD, NJ, USA) and represented as geometric shift from the mean as measured.

### 3D embedded cell cluster assay

The 96-well plates were siliconized with Sigmacote^®^ prior to cell seeding. HT1080-GFP cells were encapsulated within a 5 μl PEGylated fibrin hydrogel at 1000 cells/µl. The 5 µl droplet was pipetted in the centre of a well of a 24-well plate and allowed to polymerize for 15 min at 37°C. A 50 μl p-fibrin hydrogel encapsulating Invivofectamine^®^ 3.0 lipopolyplexes containing 500 ng Cell Death siRNA (Qiagen) was then polymerized over the 5 µl droplet. After complete polymerization, growth media was added to the well. Neg siRNA and cells only were included as controls. At either day 3 or 7, cell death was assayed by addition of EthD-1 (2 mM). Green cells (expressing GFP) were considered to be alive. Maximum intensity projections of the cell droplets were then imaged by a Zeiss LSM510 confocal microscope. Four projections encompassing the entire area and depth of the cell droplet were taken per well and the number of live and dead cells quantified by ImageJ [23].

### Animals

Handling of all mice and experiments complied with the Principles of Laboratory Care and the guidelines for the care and use of laboratory animals (NIH publication no. 86-23) and the study was approved by the Animal Research and Ethics Committee of the University of Cape Town (HSFAEC 014-022). Mice were bred and housed at the Research Animal Facility (RAF), Faculty of Health Sciences, University of Cape Town (UCT). Six- to eight-week-old (male and female) BALB/c wildtype and the BALB/c GFP transgenic homozygotes (Cby.B6-Tg (UBC-GFP) 30Scha/J) mice strains used in the study were obtained from RAF.

### Fibrin hydrogel degradation rate in vivo

Unmodified and PEGylated fibrinogen solutions (35 µl, containing Alexa Fluor™ 647 labelled fibrinogen at 1.5% m/m unlabelled fibrinogen) were prepared and injected into the tibialis anterior (TA) muscle of BALB/c mice 5 min after mixing. The mice were euthanized at 30 min, 2-, 4- and 7-days post injection. Both TA muscles per mouse were utilized and two mice were used per time point. TA muscle samples were dissected out and immediately fixed in 10% formalin (v/v). After fixation, tissue samples were processed for wax embedding and then sectioned on a microtome. Stitched fluorescent micrographs were captured on a Nikon Eclipse 90i fluorescent microscope (Nikon, Japan) with the same exposure settings used for all. Alexa Fluor™ 647 labelled fibrinogen was quantified using Visiopharm image analysis software (Visiopharm, Denmark).

### RNAi in mouse skeletal muscle

Invivofectamine^®^ 3.0 lipopolyplexes were formed with 5 µg of siGFP-Cy3 or siMstn-Cy3 and then dialysed under sterile conditions in isoPBS pH 7.4 for 2 h with a Slide-A-Lyzer^®^ Mini dialysis device kit. Mice (*n* = 16) were injected in both TA muscles with either dialysed lipoplexes alone or lipoplexes encapsulated in 30 µl PEGylated fibrin. The various groups were randomly distributed (*n* = 8). Random numbers were assigned to the groups using the random number functions in Excel with groups assigned to mice based on descending value of said random number. Mice were monitored and euthanized 7 days later. TA muscle tissue was carefully dissected out, weighed, 1 mm thick cross section excised from centre (see below) and the remainder of the explants were immediately immersed in RNAlater^®^ solution.

### siRNA retention analysis

For analysis of siRNA-Invivofectamine^®^ lipopolyplexes retention in the muscle tissue, a cross section approximately 1 mm thick was excised from the centre of the dissected TA muscle tissue and fixed in 10% formalin. After processing for wax embedding, stitched micrographs were captured on a Nikon Eclipse 90i fluorescent microscope using the same exposure settings for all images. The retention of siRNA was quantified using Visiopharm image analysis software (Visiopharm, Denmark).

### Protein and RNA extraction

The remaining portions of the TA muscle samples preserved in RNAlater^®^ had their protein and RNA extracted using a mirVana™ Paris RNA and Native Protein Isolation Kit (Thermo Fisher Scientific, MA, USA) as per manufacturer’s instructions. In brief, the tissue samples were minced with a scalpel blade into pieces on ice and then subjected to two short bursts (3–4 s) of sonication. The sample was then equally divided for total RNA and protein isolation. Protease inhibitor (PI) cocktail (1 μl/100 μl) was added to the protein isolation sample to prevent protein degradation. Total protein and RNA concentrations were quantified using Bradford reagent and absorption analysis with a Nanodrop spectrophotometer respectively. Samples were stored at −80°C until analysis for GFP expression by western blot and Mstn mRNA by RT-PCR.

### GFP western blot analysis

Protein (30 µg) from mouse tissue was electrophoresed under reducing conditions. The sample was boiled for 10 min in reducing loading dye. The proteins were then separated on a 12% SDS-page BioRad TGX™ FastCast™ Acrylamide gel. The membrane was incubated for 1 h at RT with 5% nonfat milk in Tris-buffered saline containing 0.02% Tween 20 (TBST). GFP (≈27 kDa) and the loading control β-Tubulin (≈50 kDa) protein were probed together for by Anti-GFP–ChIP Grade rabbit polyclonal (1:10 000 dilution) and anti-β-tubulin antibody (1:2500 dilution) primary antibodies in 5% nonfat milk in TBST overnight at 4°C. Protein detection was carried out using WesternBright™ECL (Advantsa) detection kit and imaging was carried out using Syngene GeneGnome XRQ Chemiluminescence Imaging System (Synegene, India).

### Myostatin quantitative real-time reverse transcriptase-PCR (RT-PCR)

After extraction and quantification of total RNA, myostatin (Mstn) mRNA expression levels were quantified using the Roche RT-PCR system. cDNA was synthesized using a Roche Transcriptor first strand cDNA synthesis kit following manufacturer’s guide. mRNA levels were quantified using LightCycler^®^ 480 SYBR Green I master mix again following manufacturers guide. The relative expression of Mstn mRNA was determined using the LinRegPCR software program [24] and normalized to GAPDH expression. GAPDH (237 bp) and Mstn (167 bp) products were confirmed by agarose gel electrophoresis.

### Statistical analysis

Normal distribution of continuous numerical data was assessed using the Shapiro–Wilk test. One-way ANOVA with post-hoc Tukey-Kramer testing for significance was performed on normally distributed data. For non-normally distributed data, the nonparametric Kruskal Wallis test followed by post-hoc Steel-Dwass testing was instead performed. *P*-values <0.05 were considered statistically significant. Analysis was performed using JMP Pro version 16 (Cary, NC, USA). Data were presented as means ± standard error of the mean.

## Results and discussion

### Stability of fibrin and p-fibrin gels and their release of siRNA-Invivofectamine^®^ lipopolyplexes

**Figure 1.**
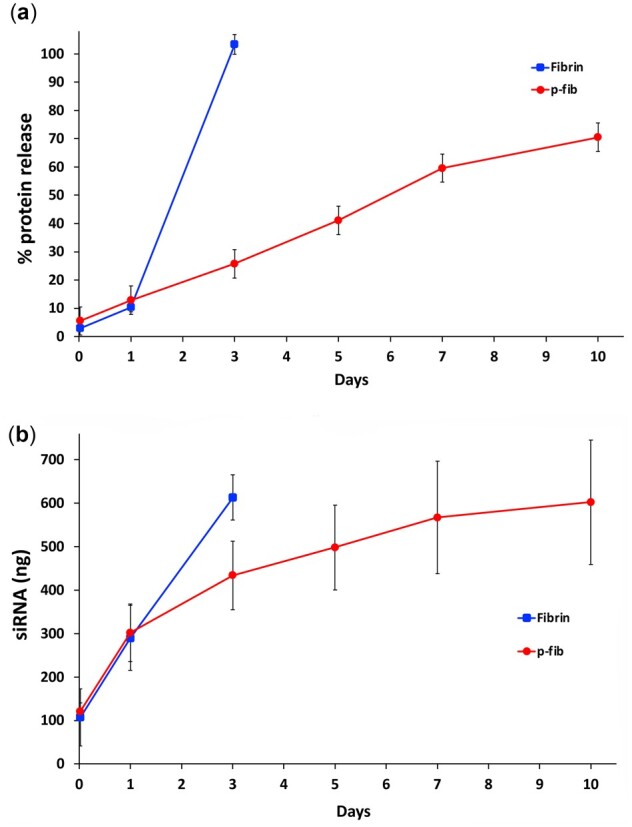
Fibrin and p-fibrin degradation rates and release of siRNA. (**a**) Degradation rate was determined by quantifying fibrin breakdown products released from hydrogels at 37°C. Three independent experiments contain three technical repeats. (**b**) siNegative-Invivofectamine^®^ lipopolyplexes were encapsulated hydrogels and eluents collected. Collected lipopolyplexes were denatured (0.5% SDS), resolved on a 2% agarose gel and the siRNA bands quantified. Three independent experiments contain three technical repeats.

A wide range of homobifunctional PEG N hydroxysuccinimide esters such as PEG-(succinimidyl methyl butanoate)_2_ and PEG-(succinimidyl carbonate)_2_ have been used to modify fibrinogen [17, 19, 25] and this type of modification has been shown to stabilize fibrin gels *in vitro* [17]. Here we used the closely related PEG-(SMC)_2_ to PEGylate fibrinogen prior to gelation with thrombin. A similar shift upwards to higher molecular weight of the bands representing the α, β and γ fibrinogen polypeptide chains to that observed before [17] was seen in this study when fibrinogen was reacted with PEG-(SMC)_2_ (Supplementary Fig. S1). The stiffness of the two hydrogels was found to be similar as reported previously for the PEG crosslinker used here [26] (fibrin and p-fibrin G′: 34.9 ± 7.5 Pa and 29 ± 5.7 Pa respectively *P* = NS). We found that fibrin spontaneously broke down completely over 3 days when incubated at 37°C in an aqueous solution (Fig. 1). This correlates closely with the 4 days observed for breakdown observed by Drinnan *et al*. [17]. It is likely that this breakdown in part reflects the presence of proteases in the fibrin gels as incorporation of aprotonin, a serine PI, greatly slows the rate of degradation (data not shown). PEGylation of fibrinogen at a 5:1 molar ratio of PEG-(SMC)_2_ resulted in the release of fibrin breakdown products up until day 10. No hydrogel was visible after day 10 which possibly reflects the fragility of a remaining crosslinked fibrin polymer. Therefore, as seen by others PEGylation in this manner resulted in increased stability *in vitro*.

Very minimal degradation of naked siRNA was found when it was incubated in fibrinogen or thrombin solutions indicating low levels of RNAses present in the major components of the fibrin hydrogel (Supplementary Fig. S2). Therefore, it was considered likely that siRNA sequences complexed with Invivofectamine^®^ 3.0 would be at least as stable as uncomplexed RNA. The ability of fibrin and p-fibrin hydrogel to control release of undegraded siRNA was determined through densitometric analysis of full-length siRNA dissociated from their lipopolyplexes and separated by agarose gel electrophoresis. Release of siRNA from unmodified fibrin gels was observed over 3 days prior to complete degradation of the hydrogel (Fig. 1). A similar amount of siRNA was released from p-fibrin gels by day 1 but thereafter a sustained release could be seen over the following 9 days. Though release of siRNA or complexed siRNA from fibrin has not been reported previously, the influence of PEGylation on protein release has been investigated [17]. The release of TGF-β1 from p-fibrin was found to be regulated by the degradation of the hydrogel in a manner that appears analogous to the siRNA release observed here with a delayed release from the p-fibrin relative to the unmodified form. Interestingly, PDGF-BB was released significantly more rapidly, and it was suggested that the TGF-β1 may have been coupled to the fibrin scaffold through the homo-bifunctional PEG crosslinkers. It is possible that the Invivofectamine^®^ 3.0 lipopolyplexes may also allow coupling to the scaffold through similar chemistry. Further, the likely larger lipopolyplexes may also be more sterically entrapped.

Therefore, PEGylation of fibrinogen both stabilized the resultant gel and sustained the release of siRNA.

### Stability of fibrin and p-fibrin in vivo

**Figure 2.**
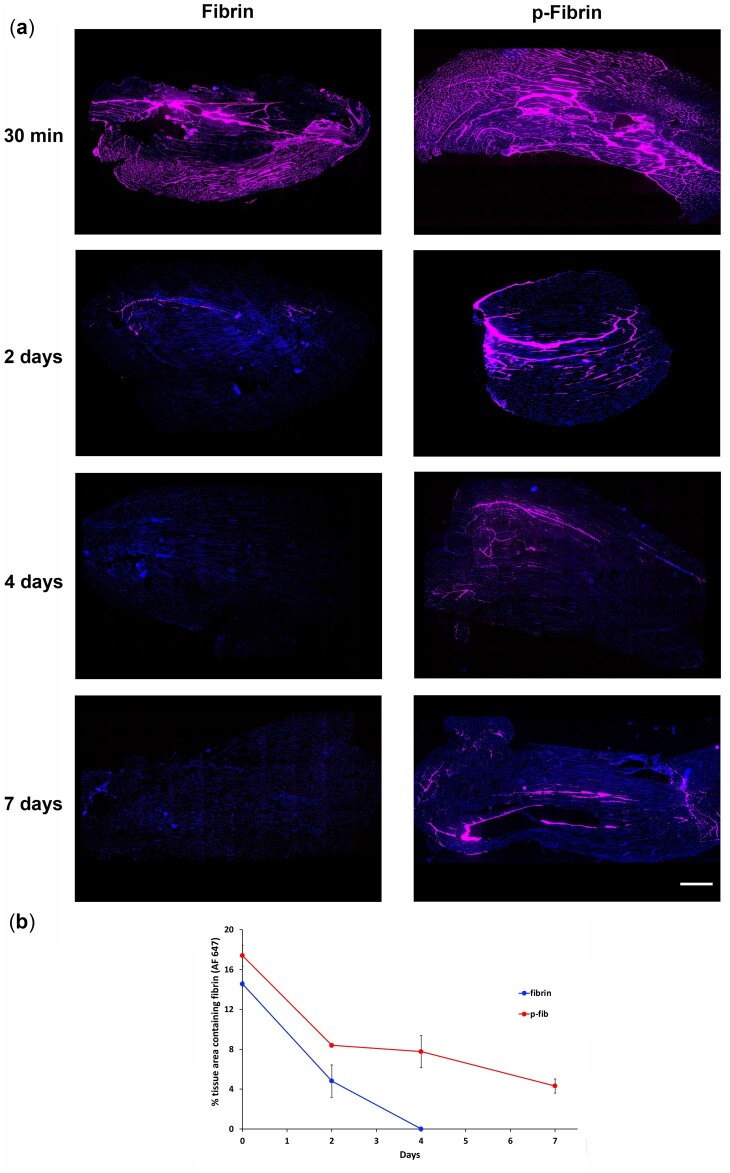
Fibrin and p-fibrin degradation in the TA muscle. Fibrin and p-fibrin hydrogels (containing Alexa Fluor 647 labelled fibrinogen) were imaged 30 min to 7 days after injection into mouse T muscle. (**a**) Stitched representative micrographs of TA muscle tissue. Nuclei (blue), hydrogel (pink). Scale bar = 500 µm. (**b**) The area of Alexa Fluor 647 labelled fibrin was quantified as a percentage of tissue area. *n* = 2 legs per group.

Though a reduced rate of breakdown *in vitro* was observed here and by others, it was not clear whether this enhanced stability would translate to the more complex *in vivo* environment. Therefore, the retention of fluorescently labelled fibrin and p-fibrin after injection into the TA muscle of mice was assayed (Fig. 2). A 30 min after delivery, the amount of labelled hydrogel was similar in both groups. However, after 2 days, there was only a slight trace of unmodified fibrin remaining and, after 4 days, no evidence of labelled hydrogel was observed. There was a much less marked reduction of the p-fibrin by 2 days, and it was clearly still present 7 days after injection. A moderate and similar inflammatory response (largely macrophage dominated) was observed for both forms of fibrin whilst present (Supplementary Fig. S3). To our knowledge, this is the first direct evidence that the increased stability of PEGylation of fibrin observed *in vitro* mirrors that seen *in vivo*. A moderate and similar inflammatory response was observed for both forms of fibrin whilst present. This finding with the sustained release of siRNA *in vitro* suggested that the release of siRNA-Invivofectamine^®^ lipopolyplexes would persist for at least 7 days after intramuscular injection when encapsulated in fibrin modified with PEG. As p-fibrin had increased durability *in vivo* accompanied with substantially lengthened period of siRNA release and limited inflammation, further analysis was focused on the crosslinked form.

### Efficacy of siRNA-Invivofectamine^®^ lipopolyplexes released from p-fibrin

**Figure 3.**
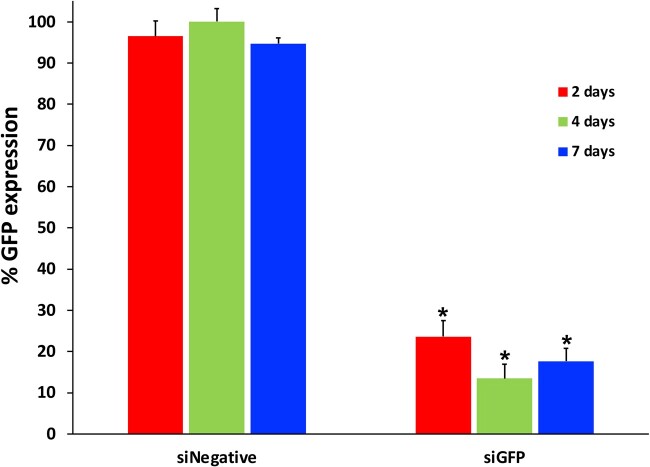
Bioactivity of siRNA-Invivofectamine^®^ lipopolyplexes eluted from p-fibrin. siRNA-Invivofectamine^®^ lipopolyplexes (siGFP or siNegative) eluted from p-fibrin hydrogels into media containing 10% serum were collected at 2, 4 and 7 days. HT1080-GFP cells were incubated with eluants in the presence of 10% serum. Post transfection, the GFP geometric mean was obtained. The GFP mean of untreated cells was used to normalize treated cells. *P<0.05 vs. siNegative-Invivofectamine^®^. Three technical repeats.

The knockdown potential of released lipopolyplexes was subsequently assessed using two complementary assays. In the first, supernatant was collected from p-fibrin containing siRNA-Invivofectamine^®^ lipopolyplexes at 2, 4 and 7 days. At all the time points, substantial and significant knockdown of GFP expression in HT1080-GFP cells was observed (76.4%, 86.5% and 82.4% GFP knockdown respectively) (Fig. 3). As the collection media contained 10% FCS, the retained efficacy indicated that the released lipopolyplexes at least somewhat protected their siRNA cargoes from prolonged exposure to RNase activity. This result also indicated that any possible coupling of the lipopolyplexes to the fibrin scaffold did not impede transfection.

**Figure 4.**
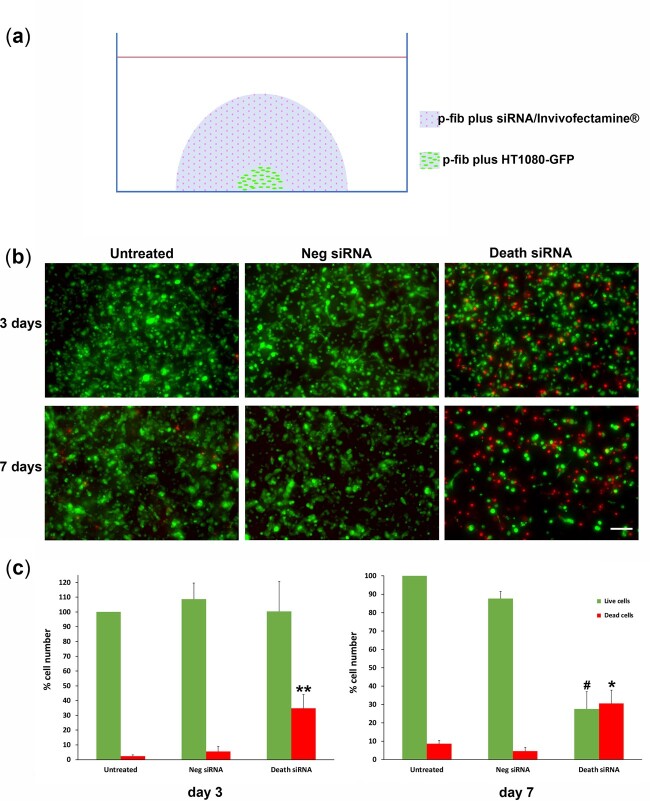
Transfection efficacy of HT1080-GFP cells in 3D embedded cell cluster assay. (**a**) Schematic figure representing the 3D embedded cell cluster assay. The outer layer contained siRNA-Invivofectamine^®^ lipopolyplexes (Death siRNA or Neg siRNA) embedded in p-fibrin with the inner region containing HT1080-GFP cells encapsulated in p-fibrin. A further condition was an outer layer that contained no lipopolyplexes (untreated). (**b**) Representative micrographs after 3 and 7 days incubation. EthD-1 was used to stain dead cells red. Green indicates viable/live HT1080-GFP cells. Scale bar = 200µm. (**c**) The number of living and dead cells for the entire inner region were counted by Image J cell counter. **P<0.01 for dead cells, * and ^#^*P*<0.05 for dead and live cells respectively (death siRNA vs. Neg siRNA and untreated). Three independent experiments contain four technical repeats.

The efficacy of siRNA-Invivofectamine^®^ lipopolyplexes embedded in p-fibrin was also assessed using a 3D assay whereby a small p-fibrin hydrogel containing GFP expressing HT1080 cells was surrounded with a p-fibrin hydrogel containing commercial siRNA sequences (complexed with Invivofectamine^®^ 3.0) that induce cell death (siDeath). This approach avoided any initial interaction of the siRNA with cells whilst in a liquid environment. After 3 days of culture, a significant increase in dead cells relative to both untreated cells and cells exposed to Neg siRNA sequences by 6-fold (*P* < 0.01) was seen in the cells encapsulated in fibrin surrounded by p-fibrin containing siDeath (Fig. 4). By 7 days, the number of live cells was also substantially reduced by 69% and the number of dead cells was significantly increased by 7-fold, relative to the negative control group. As can be seen in the images of p-fibrin injected into skeletal muscle (Fig. 2), the hydrogel is polymerized within the interstitial spaces between the muscle fibres and so an *in vitro* model, where the target cells are sequestered within a hydrogel prior to encircling it in a hydrogel containing the transfection agent, might more closely resemble the *in vivo* scenario.

### Retention of siRNA-Invivofectamine^®^ lipopolyplexes after injection into TA muscle

Both siGFP and siMstn sequences were randomly injected into the TA muscles of GFP expressing mice both alone and with PEGylated fibrinogen. It was anticipated that any changes in expression of these two genes would not influence that of the other and would thus allow for the assessment of knockdown of two genes simultaneously in one set of animals.

**Figure 5.**
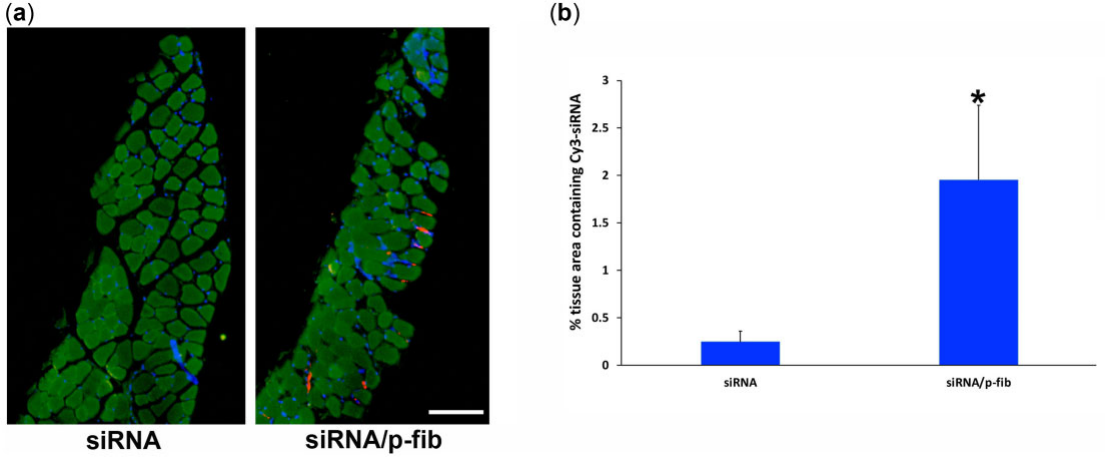
Retention of free or p-fibrin encapsulated siRNA-Invivofectamine^®^ lipopolyplexes in TA muscle tissue. Mice were randomly injected in their left and right TA muscles with p-fibrin containing siRNA-Invivofectamine^®^ lipopolyplexes made with either siMstn-Cy3 or siGFP-Cy3 (siRNA/p-fib) or with lipopolyplexes alone (siRNA). (**a**) Stitched representative micrographs of TA muscle tissue excised 7 days after delivery of siRNA-Invivofectamine^®^ lipopolyplexes. Nuclei (blue), siRNA (red). Arrow indicates an example of siRNA. Scale bar=250µm. (**b**) The area of Cy3 labelled siRNA was quantified as a percentage of tissue area. *P<0.05 vs. free lipopolyplexes group. *n*=14 and 13 for free and p-fibrin groups respectively.

Injection of siRNA lipopolyplexes in conjunction with p-fibrin resulted in a 7.9-fold increase in retention of fluorescently labelled siRNA at 7 days post-injection relative to lipopolyplexes injected alone (*P* < 0.05) (Fig. 5). In order that the maximal amount of excised tissue was preserved for downstream analysis of the outcome of RNAi (see below), a 1 mm thick cross-section from the centre of the TA muscle was excised for the above analysis. During processing and wax embedding, two samples from the lipopolyplex-alone groups and three samples from the p-fibrin/lipopolyplex groups were lost. The presence of siRNA in the interstitial regions where p-fibrin was observed previously (Fig. 2) further suggests entrapment of siRNA lipopolyplexes within the hydrogel vehicle. A similar level of mild to moderate inflammation was observed (Supplementary Fig. S4) to that seen for p-fibrin alone in the stability study described above, suggesting no pronounced effect due to the presence of lipopolyplexes.

### Influence of p-fibrin encapsulation on RNAi

**Figure 6.**
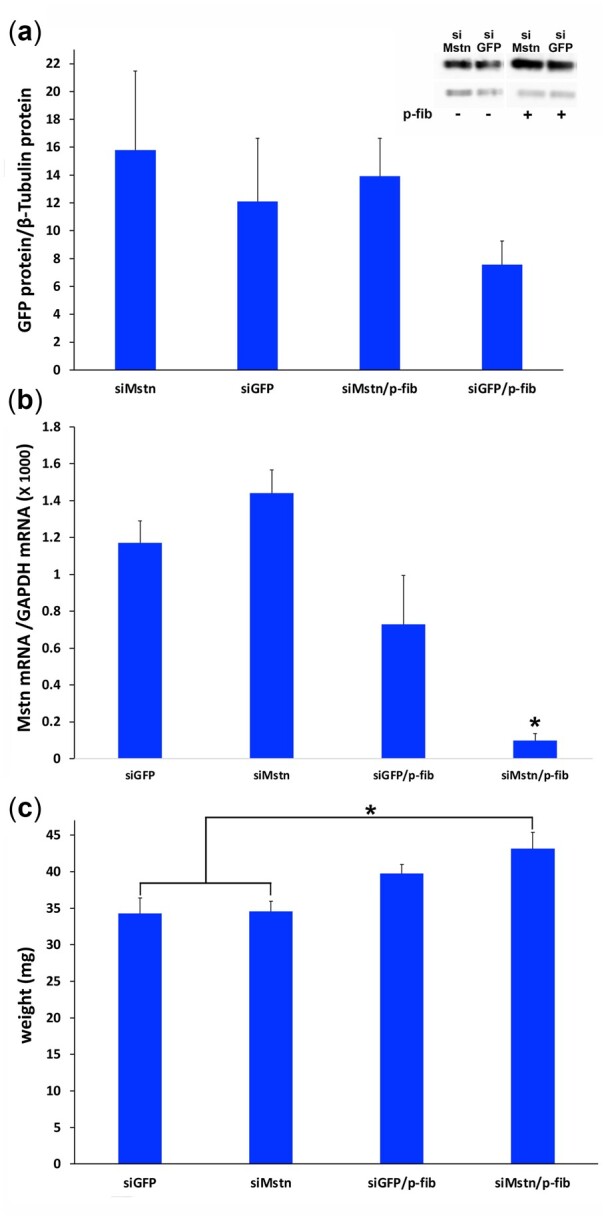
Influence of p-fibrin encapsulation of siRNA-Invivofectamine^®^ lipopolyplexes on gene expression in TA muscle tissue. Mice were randomly injected in their left and right TA muscles with p-fibrin containing siRNA-Invivofectamine^®^ lipopolyplexes made with either siMstn-Cy3 or siGFP-Cy3 (siMstn/p-fib, siGFP/p-fib) or with lipopolyplexes alone (siMstn, siGFP). Seven days post treatment TA tissue was excised, analysed for (**a**). GFP expression as determined by western blot analysis (*P*=NS) (insert: representative immunoblot lanes, see Supplementary Fig. S5) and (**b**). Mstn expression as determined by RT-PCR (*P<0.05 siMstn/p-fib vs. siGFP/p-fib, siGFP and siMstn). (**c**) TA muscles were weighed after excision (*P<0.05 siMstn/p-fib vs. siGFP and siMstn). *n*  =8.

The expression of both GFP and Mstn were then analysed at 7 days post-injection. Immunoblotting of GFP showed no significant reduction in expression of GFP after delivery of siGFP, although siGFP delivered in conjunction with p-fibrin was associated with a drop in expression of GFP relative to all other groups (37 ± 14%; 52 ± 18% and 46 ± 9% reduction vs. siGFP alone; siMstn alone and siMstn plus p-fibrin respectively) (Fig. 6).

Analysis of Mstn RNA levels by real time RT-PCR showed that Mstn expression was substantially reduced for siMstn delivered with p-fibrin against all groups (93 ± 8%; 91 ± 9% and 86 ± 31% vs. siMstn alone, siGFP alone and siGFP p-fibrin respectively (*P* < 0.05)). Only the TA muscle injected with siMstn/p-fibrin showed a significant increase in mass relative to siGFP and siMstn alone of 26% and 25% respectively (*P* < 0.05). Thus p-fibrin appears to be an effective hydrogel vehicle for entrapment and sustained release of a siRNA lipopolyplex in skeletal muscle, a challenging environment for transfection [27–29]. Interestingly, in the limited reported outcomes after siRNA mediated knockdown of GFP *in vivo*, similar nonsignificant trends in reduction of GFP expression (quantified by ELISA) were observed for GFP expressing tumours targeted systemically through intravenous injection of siRNA complexed with acid-degradable ketalized linear polyethylenimine [30]. A marked decrease in GFP silencing was also seen when siRNA was electroporated into a mouse thigh muscle 2 days after GFP expression was induced in the muscle relative to when both GFP expression plasmid and siRNA were electroporated together [31]. This might suggest difficulties in discerning knockdown effects in the context of a stable protein such as GFP and indeed even *in vitro*, a less stable variant is used to increase sensitivity to RNAi outcome [32, 33]. In a cotransfection study of an adenovirus carrying GFP and siRNA into the mouse TA muscle to investigate the efficacy of a cationic nanogel vector for the siRNA, it was argued that parallel transfection was pursued due to concerns that accumulation of protein might obscure the knockdown effect [34]. It is possible that, where siRNA against GFP was introduced into transgenic mice stably expressing GFP, such timing issues may have caused a less robust outcome. Mstn with its potential for eliciting skeletal muscle growth after inhibition has been more extensively studied in the context of RNAi. In the original study that identified the siRNA sequence used here as being an effective Mstn mRNA inhibitor, the sequence incorporated in a shRNA plasmid was electroporated in the TA of rats and the muscle excised after 2 weeks [35]. Only a 25% mRNA knockdown was achieved but this resulted in a 10% increase in muscle mass. In two related studies where the sequence was delivered with atelocollagen into masseter muscles [36] and biceps femoris [37] of mice, a 20–60% increase in muscle size was observed in the earlier study in the biceps femoris and around 35% increase in masseter mass with a 75% knockdown of mRNA at 2 weeks. In a study that examined the effect of combining mice exercise with RNAi, the siRNA sequence was delivered systemically using osmotic mini-pumps [38]. After 28 days, only the combination of siRNA and exercise resulted in an increase of the mice’s gastrocnemius muscle mass of 8%. This was associated with a knockdown of Mstn mRNA of approximately 30–40%. Interestingly in a comprehensive Cre recombinase study in mice with floxed Mstn genes, it was found that a 60% knockdown of Mstn mRNA was required to achieve an increase in muscle mass [39]. In a recent study using an alternative sequence conjugated to a highly chemically modified cholesterol, systemic administration in mice achieved 85–95% knockdown of Mstn mRNA [40]. Here an ≈8% increase in mice gastrocnemius muscle size (as determined by microcomputed tomography) was observed after 7 days. The apparent increase in mass of the TA in this present study coupled with a 90% mRNA knockdown is comparable to the findings discussed above, suggesting that delivery from p-fibrin hydrogel is advantageous. It should be noted that the level of knockdown observed *in vivo* was similar to that achieved above in the *in vitro* model, where cells were transfected with siRNA complexed with Invivofectamine^®^ 3.0 in the presence of serum.

## Conclusion

This study shows sustained siRNA delivery and RNAi *in vitro* and *in vivo* using a fibrin-based hydrogel embedded with cationic lipid-based lipopolyplexes. The degradation rate of fibrin after injection into skeletal muscle was reduced by PEGylation [17]. The transfection potential of p-fibrin encapsulated lipopolyplexes was shown to be retained with a 3D *in vitro* assay. One week postinjection into mouse skeletal muscle, the p-fibrin–siRNA–Invivofectamine^®^ lipopolyplex system could improve siRNA retention at the target site, substantially elevated Mstn gene silencing and increased muscle mass. These findings are suggestive that p-fibrin may have general utility for controlled delivery of lipopolyplexes carrying RNAi agents.

## Supplementary Material

rbac101_Supplementary_Data
