# Supplementary material for: Modified fibrin hydrogel for sustained delivery of RNAi lipopolyplexes in skeletal muscle
Source: Regen Biomater. 2022 Dec 13;10:rbac101. doi: 10.1093/rb/rbac101 (PMC9887344; doi:10.1093/rb/rbac101)
Supplement: rbac101_Supplementary_Data [file rbac101_supplementary_data.docx]

**Supplementary data**

*Table 1: Primer sequences used in RT-PCR analysis*

| Primer gene target name | Forward Primer | | Reverse primer | Concentration  (nM) | |  |
| --- | --- | --- | --- | --- | --- | --- |
| Myostatin | 5’-CAGCCTGAATCCAACTTAGG-3’ | 5’-TCGCAGTCAAGCCCAAAGTC-3’ | | | 250 | |
| GAPDH | 5’ -TTCACCACCATGGAGAAGGC-3’ | 5’ -GGCATGGACTGTGGTCATGA-3’ | | | 500 | |

*Table 2: siRNA sequences used in polyplexes*

| siNegative - Negative scramble control siRNA, Bioneer Corp (Daedeok District, South Korea). Sequence not available. |
| --- |
| siGFP - Green fluorescence proteins siRNA: GCAUCAAGGUGAACUUCAAdTdT (sense), Bioneer Corp (Daedeok District, South Korea). |
| Neg siRNA - AllStars Hs Cell Death scramble negative control siRNA, Qiagen (MD, USA). Sequences not available. |
| Death siRNA - AllStars Hs Cell Death positive control siRNA (1027299), Qiagen (MD, USA). Sequences not available. |
| siNegative-Cy3 - AllStars negative Scramble Cy3 labelled siRNA (AF555), Qiagen (MD, USA ). |
| siGFP-Cy3 - Cy3 labelled siGFP: GCAUCAAGGUGAACUUCAAdTdT Cy3 (sense), GE Healthcare Dharmacon. |
| siMstn-Cy3 - Cy3 labelled siMstn: AAGAUGACGAUUAUCACGCUA-dTdT-Cy3 (sense), GE Healthcare Dharmacon. |
| Fibrinogen (human serum); thrombin (bovine plasma), Sigma Aldrich, MO, USA. |
| PEG (Succinimidyl Carboxymethyl Ester)2 MW3500 (PEG-(SMC)2), JenKem Technology, TX, USA. |
| Alexa Fluor™ 647 conjugated fibrinogen (human plasma); Alexa Fluor® 660 C2-maleimide; ethidium homodimer-1 (EthD-1) stain from Live/Dead™ cell viability assay, Thermo Fisher Scientific, MA, USA. |
| Invivofectamine® 3.0; mirVana™ Paris RNA and Native Protein Isolation Kit and RNAlater® Solution, ThermoFischer Scientific, MA, USA. |

**Supplementary figure 1: PEGylation of fibrinogen at 5:1 PEG to fibrinogen molar ratio**. 10 mg/ml fibrinogen was either pegylated or not with succinimidyl carboxymethyl ester derivative of PEG (PEG-SMC_2_). 5µl (125ng of fibrinogen protein) of the fibrinogen was added to 8.3μl 3X loading dye with 15% β-mercapthoethanol and volume made up to 25μl with distilled water. Samples were boiled for 10mins, loaded onto a 7.5% SDS PAGE BioRad TGX™ FastCast™ Acrylamide gel and electrophoresed at 200 V for 45mins. After electrophoresis fibrinogen protein on the gel was stained by coomassie stain for 30mins and distained for 2hrs with mild shaking at RT.Image of SDS-PAGE gel stained for protein with Coomassie Brilliant Blue R-250. M lane=molecular weight marker in kDa. Lane 1 is unmodified fibrinogen. Its PEGylation with PEG-SMC_2_ was at ratios 5:1 (lane 2). Image is a representation of n=2.


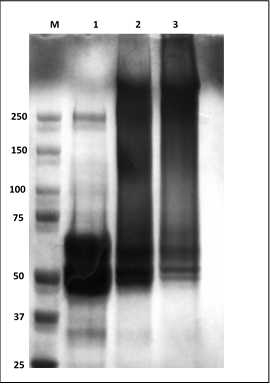


**Supplementary figure 2: Effects of fibrin gel components (fibrin and thrombin) on siRNA degradation over time.** 200ng of siNegative was incubated over 5-day period with fibrinogen (10 mg/ml final concentration) in HBS buffer and 0.624U/ml thrombin in 40mM CaCl_2_ HBS buffer. Fibrinogen, thrombin and fresh 200ng siRNA were included in the 2% agarose gel electrophoresis as controls. Throm: thrombin. Image is a representation of an experiment with three technical repeats.


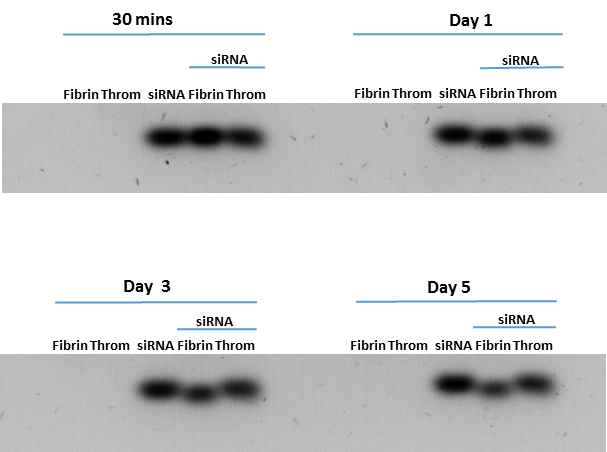


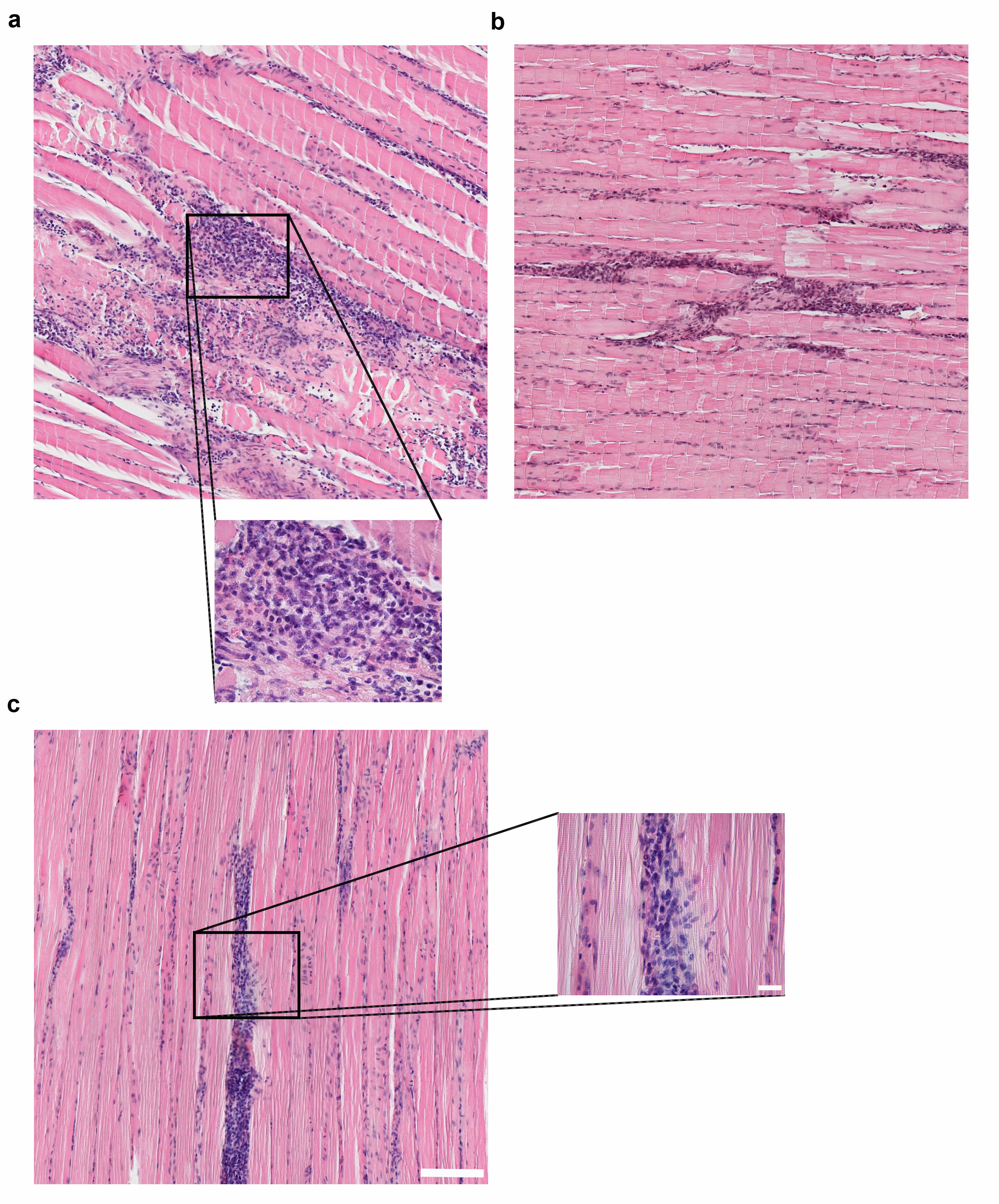


***Supplementary figure 3: Hematoxylin and eosin stain of mouse tibialis anterior injected with fibrin or p-fibrin. a.*** *2 day explant of fibrin* ***b.*** *2 day explant of p-fibrin* ***c.*** *7 day explant of p-fibrin. Scale bar 100 µm. High magnification micrographs: Scale bar 20 µm*


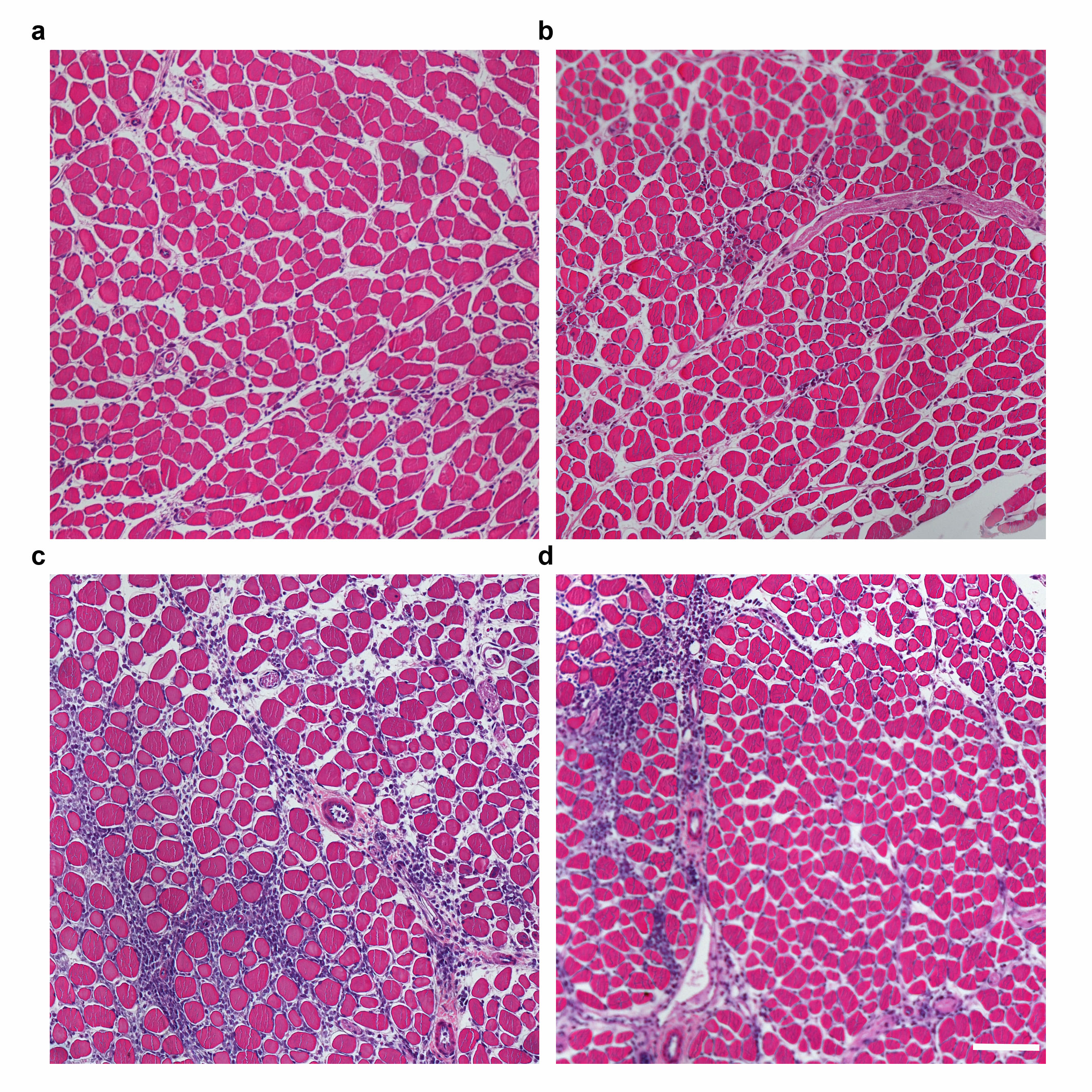


***Supplementary figure 4: Hematoxylin and eosin stains of mouse tibialis anterior injected with lipopolyplexes with/without p-fibrin after 7 days.*** *a. siGFP-Cy3 alone b. siMstn-Cy3 alone c. siGFP-Cy3/p-fibrin d. siMstn-Cy3/p-fibrin. Scale bar 100 µm.*


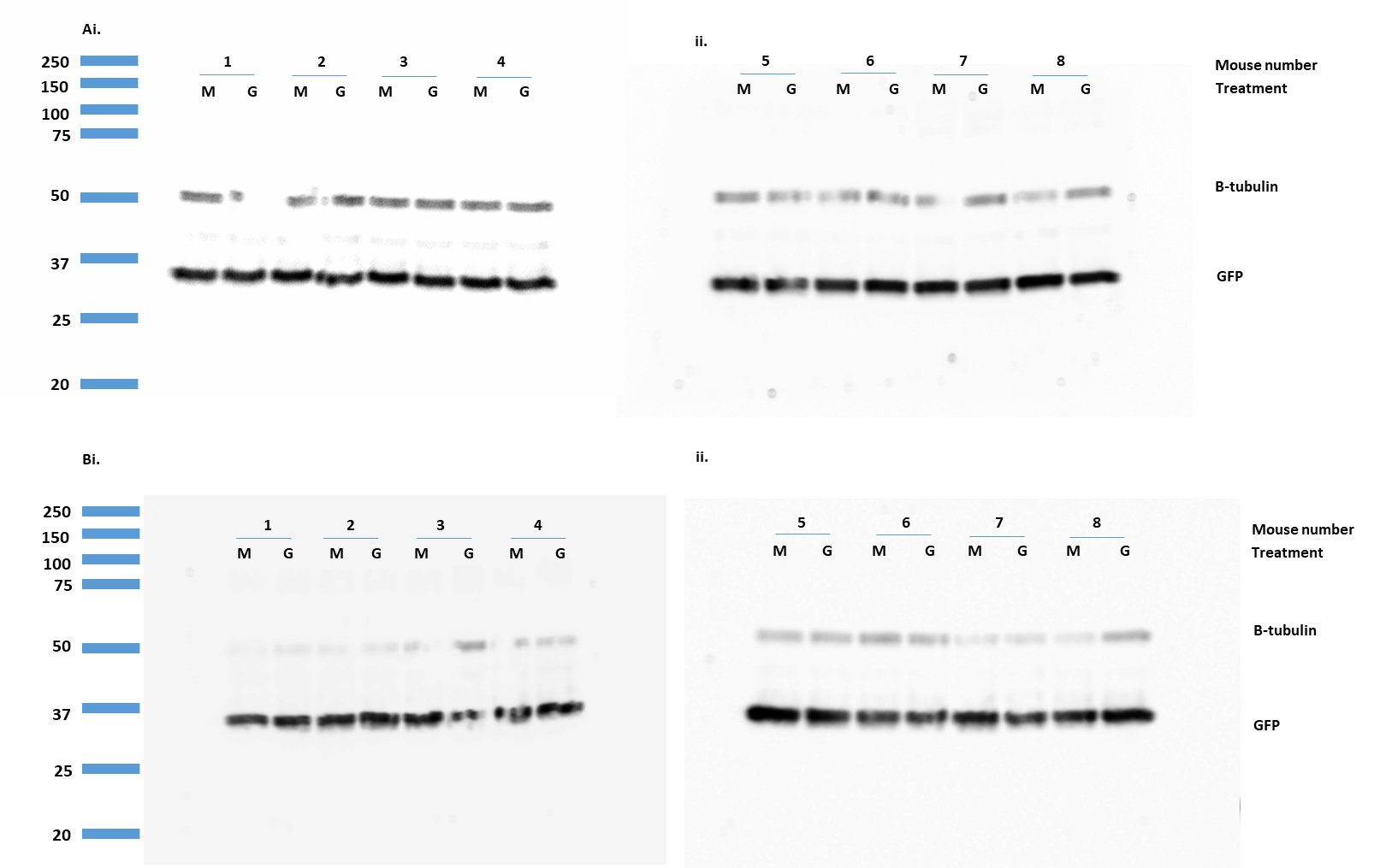


**Supplementary figure 3: Influence of p-fibrin encapsulation of siRNA-Invivofectamine® lipopolyplexes on GFP expression in TA muscle tissue.** Mice 1-8 were randomly injected in their left and right TA muscles with either siMstn (M) or siGFP (G) lipopolyplexes alone (**Ai-ii)** or with p-fibrin containing siRNA-Invivofectamine® lipopolyplexes made with either siMstn-Cy3 or siGFP-Cy3 (siMstn/p-fib, siGFP/p-fib in **Bi-ii**). 7 days post treatment TA tissue was excised, analysed for GFP expression as determined by western blot analysis. GFP levels densitometry quantification was normalised to β-Tubulin. N=8 in each experimental group. MW = Precision Plus Protein™ Dual Color Standards BioRad.
